# Natural Savanna Systems Within the “One Health and One Welfare” Approach: Part 2—Sociodemographic and Institution Factors Impacting Relationships Between Farmers and Livestock

**DOI:** 10.3390/ani15142139

**Published:** 2025-07-19

**Authors:** Marlyn H. Romero, Sergio A. Gallego-Polania, Jorge A. Sanchez

**Affiliations:** 1 Department of Animal Health, Faculty of Agrarian and Animal Sciences, Universidad de Caldas, Manizales 170004, Colombia; jorge.sanchez@ucaldas.edu.co; 2 Veterinary Research Group, Faculty of Agrarian and Animal Sciences, Universidad de Caldas, Manizales 170004, Colombia; sergio.gallego25728@ucaldas.edu.co

**Keywords:** women’s empowerment, generational change, governance, human–animal relationships

## Abstract

The relationships between farmers and livestock are multifaceted. The aim of this study was to describe the sociodemographic, biogeographic, and institutional factors that influence the relationships between humans and animals in the natural savanna. Visits were made to 65 farms, followed by interviews (*n* = 13) and three focus group interviews (*n* = 24) directed at farmers and institutional representatives. The results were triangulated to extract the key findings. The following findings were obtained: (a) cultural gender transitions and the lack of generational succession have transformed livestock farming; (b) the relationships between farmers and livestock have favored the implementation of new productive practices and innovations, as well as improvements in animal welfare practices; (c) conditioning factors affecting these relationships include gender discriminatory norms, low profitability and credit access, poor sanitation, animal handling infrastructure, security, and resistance to change; and (d) improvement opportunities include the inclusion of young people and women in livestock farming, education for work practices, credit facilitation, access to technologies, governance, and improvement in the cattle logistics chain. The results are useful for enhancing the relationships between farmers and livestock, guiding training activities, and responsible governance.

## Simple Summary

Cattle management is a representative aspect of animal welfare. The aim of this study was to describe the sociodemographic, biogeographic, and institutional factors that influence the relationships between humans and animals in the natural savanna. A mixed-methods study (using qualitative and quantitative methods) was conducted on 65 commercial farms located in the department of Vichada. Changes were observed in women’s participation in livestock farming and in generational succession, which have fostered the adoption of animal welfare practices and production innovations. Barriers to change were identified, such as the invisible and undervalued contribution of women, institutional challenges (basic sanitation, security, infrastructure, and pre-slaughter logistics), and resistance to change. Opportunities for improvement included the empowerment of women and young people in rural areas, integrative methodologies, access roads and marketing channels, and responsible governance. There is a need to integrate social sciences and the humanities to better understand human–animal relationships and to promote more inclusive and sustainable agricultural policies.

## 1. Introduction

Rural areas are defined not only by their physical characteristics but also by the features of the social life that evolves within them [1]. Rural activities associated with livestock production can provide life stability, social acceptance, a healthy natural environment, and food security and are seen as part of cultural heritage [2]. Social changes have transformed rural lifestyles, generational transfer dynamics, productive and occupational roles based on gender identity, the make-up of households, and environmental challenges [3,4].

The relationship between people and animals is ancient, mutually beneficial, and dynamic, influenced by factors considered essential to the health and well-being of both [5]. These factors are as follows: (a) the characteristics of the people responsible for their care and handling (beliefs, opinions, attitudes, personality, identity, feelings, empathy, sociodemographic factors); (b) external factors (sociocultural context, traditions, economic aspects); (c) occupational factors (working conditions, job satisfaction and environment, psychological and social factors, accidents and injuries, effects of chronic exposure to physical risks); (d) social sustainability (employment and payment practices, associativity, health and safety conditions, job training, community relations, and environmental governance); and (e) the requirements of national and international health legislation, among other aspects [5]. Likewise, the well-being of livestock handlers is an important component of the social aspect of sustainability in the beef supply chain [6].

This study is part of a broader project aimed at “Developing a proposal for a model that integrates human welfare, animal welfare and environmental indicators to evaluate cattle breeding production systems in natural savanna, with a One-Welfare concept”. The results of the present study complement those previously presented in the following article: Natural Savannah Systems Within the “One Welfare” Approach: Part 1. Traditional Farmers’ Perspectives, Environmental Challenges and Opportunities **[7]**. We evaluated the perceptions of human, animal, and environmental well-being among cattle farmers in the natural savanna of Vichada. We found that cattle, their welfare, and environmental care are all part of farmers’ culture and pride in “being good farmers.” Animal welfare is perceived as the care of the animal’s body (good nutrition and health), protection from adverse environmental conditions (appropriate environment), and ensuring the animal’s natural behavior [7]. It was also found that human–animal relationships are mediated by multiple factors, which are evaluated in this second article and are consistent with those described by Losada-Espinosa [5].

The human–animal relationship has mainly been studied through indicators such as behavioral changes (frequency of vocalizations, body postures, facial expressions, movement patterns, and the animal’s response in the presence of humans), physiological indicators (heart rate, body temperature, hormone levels) and others associated with animal welfare (body condition, coat condition, presence of wounds or injuries, the animal’s ability to express natural behavior), and indicators of a rewarding experience (pleasure, relaxation) [8], as well as measurement scales evaluating human–animal interaction and human attitudes and empathy toward their peers and animals under their care [9]. However, less attention has been given to studying the relationship between cattle producers and their livestock, as well as the sociocultural factors and other aspects of human well-being that shape it [10]. The daily care, health, and productivity of the animals are the focus of cattle farmers’ work; their interactions are always mediated by the relationship the farmer maintains with their work within a specific social environment [11].

The sociological approach remains somewhat underestimated when it comes to improving the standards of animal care associated with handlers’ changing attitudes [8]. Social research focuses on the nature of the relationship between cattle producers and animals in terms of emotional construction and how farmers become emotionally attached to or detached from their animals [12]. Livestock handling is itself a representative aspect of animal welfare, where the physiological, behavioral, and emotional states of animals are managed by a human caregiver. It therefore requires a set of deep and complex skills to be carried out properly [13].

Given the growing interest in behavioral change among farmers in relation to animal welfare, and as a continuation of the first published article (part 1) [7], the objective of this study was to describe the factors that have transformed cattle ranching and altered the relationships between farmers and animals. This study contributes to the identification of factors that may affect the relationships between farmers and their livestock in natural savannas.

## 2. Materials and Methods

### 2.1. Ethical Considerations

This research was reviewed and approved by the Ethics and Animal Experimentation Committee of the Faculty of Agricultural Sciences of the University of Caldas- Activities with minimal risk (Act 01/08/18/2023), and informed consent was obtained to participate in this study. Participants were informed about the objectives, methods, and implications of this study for them and the field of study; likewise, participants were made aware of their right to choose not to answer any questions.

### 2.2. General Description

In Colombia, the Altillanura is a subregion of the Orinoquía, located in the eastern plains of the Andes Mountain range, extending over the Orinoco River basin. The department of Vichada is the largest in the Altillanura in terms of land area, with 78% of the land consisting of natural savannas used for extensive livestock production (4.2 million hectares) [14]. This study was conducted in the natural savanna of the Vichada department on farms located in the flat and dissected Altillanuras, which mainly differ in topography and drainage. The former is characterized by extensive and relatively uniform terrain (flat or slightly undulating), with deep, well-structured, highly porous, and permeable soils, with good natural drainage, although it may have slightly depressed, concave zones with poor drainage. The dissected Altillanura, on the other hand, has more rugged topography and landscapes shaped by erosion and more complex and heterogeneous drainage [14]. However, in this research, no differentiation was made between farms or participants in the qualitative study according to their location in the type of Altillanura, as the focus was on the social and cultural group (llanero) and the concept of “being a good farmer”.

A multistage mixed-methods approach was used, which included visits to cattle breeding farms in natural savannas, as well as the application of structured surveys [15] in-depth interviews, and focus groups.

### 2.3. Quantitative Study

#### 2.3.1. Structured Survey and Sampling

We designed a survey based on the current literature and our experience, which was pre-tested by five veterinarians to assess indicator clarity and refined based on their feedback. The survey consisted of 54 variables related to the quality of life: 7 sociodemographic variables, 13 indicators of material human well-being (housing, access to technologies, economic income, employment, expenses and consumption, economic deprivation), 27 indicators of non-material conditions (health, work–life balance, education, social fabric, and safety), and 7 indicators related to subjective conditions (cognitive, affective, and eudaimonic well-being) [16]. In addition, a questionnaire was administered to assess agroecological indicators and a validated animal welfare protocol, the results of which will be the subject of a separate publication.

Stratified probabilistic sampling was conducted based on the breeding farm census in the municipality of La Primavera, which accounted for 45% of the departmental bovine population (*n* = 130,552 animals), distributed across 29 rural districts (*n* = 657 farms), with the most representative in terms of cattle inventory being Santa Barbara (16.6%), Nueva Antioquia (15.8%), Matiyure (9.3%), and El Triunfo (5.8%) [17]. A total of 65 farms were visited, proportionally distributed among the following four municipalities: (a) Santa Barbara (*n* = 24), (b) Nueva Antioquia (*n* = 21), (c) Matiyure (*n* = 12), and (d) El Triunfo (*n* = 8). A pilot test was conducted on twelve natural savanna breeding farms to evaluate the applicability and acceptance of the protocol. The pilot test was carried out by a single trained observer (a SAG doctoral student) familiar with the region, who applied the structured survey for data collection. Once the instrument was applied, information was systematically analyzed, areas for improvement were identified, the clarity of the questions and participants’ understanding were evaluated, questions were adjusted taking into account participant feedback, and necessary modifications were made. These evaluations were not included in the final database to avoid biases from including data on variables that needed to be adjusted or eliminated. In our case, variables related to income, expenses, and safety were modified.

#### 2.3.2. Data Analysis—Quantitative Study

Descriptive analyses and frequency tables were used to explore data distribution. Unconditional associations between variables were tested using Pearson’s Chi-squared or Fisher’s exact test for categorical variables. All statistical analyses were conducted using Stata 17 software (StataCorp, College Station, TX, USA) [18].

### 2.4. Qualitative Study (Focus Groups and Interviews)

Three in-person discussion groups (male cattle farmers, female cattle farmers, and institutional representatives) were formed in the municipality of La Primavera, as described by Romero et al. [7]. Two groups of farmers (male and female) were formed based on whether the farmers participated in the farm as managers and/or owners. These groups were separated to avoid biases related to non-response or responses influenced by social norms [19]. Additionally, 11 semi-structured interviews were conducted based on the quantitative study results, targeting farmers (men and women) (*n* = 7) in the natural savanna (Vichada) and institutional representatives (*n* = 4): the Secretary of Agriculture and Rural Development of the Municipality of La Primavera, the director of Cormacarena, the Coordinator of the Colombian Cattle Farmers’ Federation in Vichada, and the President of the Cattle farmers’ Association of La Primavera. It is important to note that those who participated in the focus groups were not the same individuals who were interviewed.

Participants in in-person interviews were selected using purposive sampling [19], according to the following inclusion criteria: persons ≥18 years old, having resided/worked in the municipality within the last five years, and having substantial knowledge of livestock management in the region (required for institutional representatives). Since little is known about the ideals and identities of cattle farmers and institutional workers regarding the concept of human, animal, and environmental well-being from the One Welfare perspective and about the social changes and factors influencing how farmers relate to cattle, a qualitative approach using semi-structured interviews and focus groups was appropriate for our study [20]. The interviews were conducted by one of the authors and a sociologist specialized in qualitative research techniques and were audio-recorded with informed consent. The interview guide is included in the Supplementary Materials S1.

#### Data Analysis—Qualitative Study

Focus group dialogs were professionally transcribed and compared with the original audio recordings for accuracy. Scripts were read the transcripts and conducted initial coding using a bottom-up inductive approach to identify potential themes and subthemes. Then, they reviewed the codebook, themes, and subthemes, adjusting until consensus was reached. Participants were anonymized, and quotes were coded to illustrate the key elements of the themes. Selected quotes exemplified specific categories within each theme, highlighting representative statements. The findings from both the qualitative and quantitative studies were triangulated and are presented in an integrated form in the Results Section.

Thematic analysis was used to identify, analyze, and report patterns within the written data using NVivo software (version 10.2.2; QSR International, Burlington, MA, USA).

### 2.5. Positionality and Reflexivity Statement

The authors’ positionality was guided by the previously described framework [7].

### 2.6. Data Presentation

**Figure 1:**
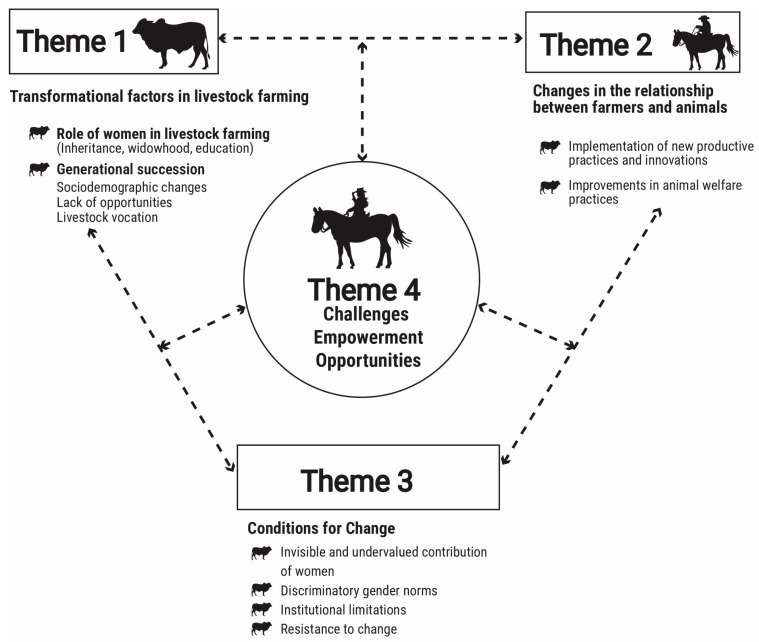
A thematic map summarizing the results of the mixed-methods study and describing the factors that have shaped human–animal relationships in the natural savanna of La Primavera, Vichada.

A thematic map (Figure 1) was produced, and textual quotations were reported as examples of themes and identified by participant numbers. Clarifying text is included in parentheses; brackets were used when a word was changed to avoid using slang language or to preserve the identity of the participant, and ellipses were used to indicate a part of the quote that was removed for brevity and did not change meaning.

## 3. Results

### 3.1. Demographics

A sociodemographic description of participants in both the quantitative and qualitative studies is presented in Table 1. The majority of participants were men, with primary education and more than 30 years of experience.

### 3.2. Key Themes Identified

The relationships between humans and animals in the natural savanna have been shaped by factors associated with gender transitions and generational shifts (Theme 1. Factors transforming livestock production), which have in turn strengthened human–animal bonds (Theme 2) through the adoption of new production practices and improved animal welfare measures. However, constraints to change still exist, such as the invisible and undervalued contribution of women, institutional challenges (basic sanitation, security, infrastructure, and pre-slaughter logistics), and resistance to change (Theme 3).

Identified opportunities for improvement (Theme 4) include the empowerment of women and rural youth, integrative methodologies, improved access routes and marketing systems, and responsible governance.

#### 3.2.1. Theme 1: Transformational Factors in Livestock Farming

Livestock farming and working conditions for farmers have changed in recent years, as the activity has faced economic, social, environmental, and ethical challenges. Participants identified several factors that have driven and transformed livestock activity in the natural savanna, with both positive and negative impacts on their work and cultural identity.

a. Changing Role of Women in Livestock Farming

Livestock farming has traditionally been a male-dominated activity in the natural savanna, shaped by stereotypes of physical strength and toughness in handling cattle. Consequently, women’s roles have been associated with tasks that do not require physical force. Traditionally, women have been responsible for managing the household, raising children, caring for backyard animals (laying hens, pigs, sheep), milking, cheese-making, and maintaining the garden and vegetable patch. However, as noted by both male and female farmers and institutional representatives who participated in discussions and interviews, women today are increasingly involved in administrative activities and cattle management due to factors such as inheritance, widowhood, education, and a love for livestock farming (Table 2).

b. Lack of Generational Succession

Participants expressed concern about the abandonment of traditional livestock farming, as 78.4% of respondents were over 44 years old (see Table 2), which could hinder the transmission of cultural knowledge to younger generations. Other contributing factors to the lack of generational succession include rural living conditions (lack of opportunities, limited access to education, absence of vocational interest in ranching) and changing ideals among youth. This last aspect was considered significant, as expressed by participant [P32_GR1M]: “The herds really began to disappear when the children left to study. Some returned, but many others left the countryside and never came back. The parents didn’t encourage the idea of returning”. The widespread absence of the generational transfer of cultural knowledge has led to a decline in traditional cattle herding songs (Table 2).

#### 3.2.2. Theme 2: Changes in the Relationship Between Farmers and Animals

Gender dynamics, access to education, and generational succession are central to creating sustainable and inclusive agri-food systems. Farmers and institutional participants identified changes that have strengthened relationships between farmers and their animals, as outlined below:

- Implementation of New Productive Practices and Innovations

New productive practices have been gradually introduced in the territory through collaboration with government institutions responsible for animal health and welfare, vocational education for young male and female farmers, and increased female involvement in farm administration, as stated by [P21_GR1H]: “Previously people didn’t invest anything in the farm, now some farms are improving their breeds, improving pastures [...] Management consists of having well-prepared paddocks, with shade and water sources...” (Table 3).

Institutions have contributed positively to implementing new practices aimed at mitigating the impact of global warming and strengthening good ranching practices, artificial insemination, pasture management, animal health, and solid waste management, among other aspects. Among innovations, participants mentioned professional assistance, although it is still limited, as expressed by [P8_GR1M]: “... there should be a veterinarian in each region advising us, but sometimes finding a vet here is difficult.”

However, the sociological urbanization of rural areas and the introduction of technologies unfamiliar to tradition have disrupted cultural significance, transforming llanero identity—for example, the use of motorcycles instead of horses to herd cattle. While seen as efficient over large areas, this change affects traditional skills and cultural heritage, as explained by farmer [P19_GR2M]: “Yes, of course, the tradition is lost because they use motorcycles. The horse, on the other hand, is a form of wisdom.”

b. Improvements in Animal Welfare Practices

The cattle ranching culture in the natural savanna is not static. On the contrary, it constantly evolves through complex economic and social processes that transform certain practices, beliefs, and roles. The participation of university-educated male and female farmers has enhanced relationships with animals and led to more effective welfare practices. The role of women in livestock farming is also a driver of change with important implications for cattle farming in Colombia and other countries, although this process is still in its early stages.

In focus groups, participants highlighted that on farms where women are recognized and actively involved in ranching, they show a strong ability to adapt to new challenges, implement sanitary regulations, and transfer knowledge to their children. Women serve as the emotional support of their partners and families, take on administrative and decision-making roles, and lead positive changes in animal management and environmental care, as shared by participant [P14_GR2W]: “The local people have learned a lot because companies have come in with new livestock rearing models. In my case, I have implemented good animal management at home and I tell my husband and the man who helps us: do not mistreat the animals. We haven’t had any problems; and we’ve learned a lot from several people who came and talked to us about it.”

Women and young farmers are showing a trend of greater inclusion and active participation, taking on a social commitment to these issues. They also participate in financial decision-making on farms, thanks to their training, as participant [P7_GR2W] stated: “Since I was very young, I was taught to be a woman with a passion for livestock farming, but passion requires two fundamental things: supporting the farm and the family economically.” In terms of gender, women’s roles are notable in public speaking, community participation, and administrative areas (Table 3).

#### 3.2.3. Theme 3: Conditions for Change

Farmers (men and women) and institutional representatives identified several factors that shape relationships between farmers and animals in the natural savanna. Below are the most relevant conditions as reported by participants.

- Invisible and Undervalued Contribution of Women

Women’s roles in livestock farming are multifaceted, but their work is still undervalued. There is income inequality, or no compensation compared to their male partners, fostering economic dependency. Their functions, responsibilities, challenges, and opportunities are also not adequately recognized. Likewise, credit access and borrowing capacity are limited (Table 4).

b. Discriminatory Gender Norms

Gender norms are socially accepted rules defining behaviors and expectations based on assigned gender at birth. Worldwide, these norms often assign submissive and reproductive roles to women and authoritative, productive roles to men. This perspective also exists in the savanna, where ranching is traditionally framed within a masculine strength stereotype, undervaluing women’s roles at home and in animal care. As one farmer [P112_INTERM] put it, “Yes, there is machismo, but more than that, we men worry about women’s safety in cattle handling because it requires strength... Some women are strong, but they’re rare exceptions.”

Women’s contributions are also under-recognized at the institutional level. Training and outreach programs are often directed at men, although women have expressed a clear desire for greater participation and recognition of their abilities (Table 4).

c. Low Income and Limited Access to Financing

While livestock farming can be a fulfilling vocation, it is also extremely challenging, requiring skills and adaptability. Improvements such as better infrastructure, food availability for animals, climate change mitigation practices, and public services in homes require financial investment and high profitability. This study found that informal employment is prevalent in the region (52%), with no formal contracts, social security, or benefits. Additionally, 35.4% of farmers relied on verbal work agreements. This limits resources for investment and reduces opportunities for achieving economic well-being for families. These conditions reduce investment capacity and economic well-being. Credit and borrowing opportunities are also limited (Table 4).

d. Institutional Limitations

Basic sanitation in the region is poor. Access to drinking water is unequally distributed, excreta disposal is via septic tanks, there is no sewage system, and solid waste collection is deficient in rural areas. Both people and animals consume water from natural sources, and most cooking is conducted with firewood. About 69.2% (*n* = 45) of farmers are affiliated with the subsidized healthcare system and 20% (*n* = 13) with the contributory system, and 10.8% (*n* = 7) lack healthcare and rely on private services. These conditions affect human well-being and indirectly influence the adoption of good animal welfare practices due to the close connection between farmers and their animals.

Colombia endured over 50 years of armed conflict between the government and FARC guerrillas, officially ending with the 2016 peace agreement. However, dissident groups and illegal actors persist, engaging in kidnapping, extortion, cattle theft, and illegal fees (“vacunas”) that create uncertainty among producers.

Infrastructure in the region is lacking, affecting pre-slaughter logistics (moving, sorting, and loading animals for transport to markets or slaughterhouses). Access to the savanna is by road or river, depending on the rainy season. Roads are only partially paved; river transport is expensive but widely used for moving food, supplies, and animals. Fattening cattle are often moved on foot, in journeys lasting up to 8 days.

Farmers do not receive state subsidies, and there is little public investment in roads, healthcare, technical assistance, or services that support productive systems. The authorized slaughterhouse is far from productive zones, farms lack equipment to weigh cattle, and transportation infrastructure is inadequate.

e. Resistance to Change

Farmers expressed that some sanitary practices mandated by national legislation contradict animal welfare principles. For example, frequent vaccination campaigns for notifiable diseases (e.g., foot-and-mouth disease, tuberculosis, brucellosis) require gathering animals often, which increases stress and disease susceptibility, lowers milk production, and reduces weight gain. As participant [P8_GR1] said, “Vaccinating all the time makes you waste time working with the cattle and trying to get a vet.” (Table 4).

#### 3.2.4. Theme 4: Challenges and Opportunities for Improvement

- Empowerment of Women and Rural Youth

Involving women and ensuring generational succession can boost the profitability of production systems by increasing the number of people engaged in ranching—people more open to change and adopting sustainable practices. However, this work must be compensated fairly and time for leisure allowed, as caregiving duties can otherwise become overwhelming.

Creating communication channels between institutions and traditional farmers fosters educational and professional interactions that influence practices and decision-making. It is essential for young men and women to have better access to credit and participate institutionally to help shape improved public policies in the region (Table 5).

b. Integrative Pedagogical Methodologies

Transforming rural lifestyles involves complexity that can be addressed through educational processes promoting social and gender equality. These include improved access to information, the adoption of new technologies, increased literacy, and fewer economic barriers to changing and implementing better, more sustainable (environmental, social, and economic) livestock practices.

Access to electricity and technology can support change and learning. As [P6_GR3I] said, “If we had electricity and access to technology, our work could be more efficient [...]”. These strategies should include both young and experienced individuals, who can preserve valuable traditions while contributing to change. As [P38_GR3] shared, “Education will bring change, but it won’t be immediate or easy, because there’s a cultural stereotype—the ‘llanero’ type—and people think if they don’t act that way, they stop being llaneros.”

c. Access and Marketing Logistics

Improved cattle transport logistics would enhance animal welfare, reduce occupational risks for handlers, lower economic losses (due to mortality, bruises, poor meat quality), increase producer incomes, and improve the regional meat industry’s reputation. Although infrastructure projects have been implemented, they remain insufficient. It is necessary to strengthen the cattle logistics chain by building slaughterhouses, cold chains, and integrated marketing systems for refrigerated meat to reduce the need to transport live animals.

d. Responsible Governance

Governance in the savanna must be strengthened to facilitate coordinated and integrated decision-making among all levels of government (municipal, departmental, regional, national), public and private institutions, social actors, and the production sector. This is vital to address regional challenges, improve public health, ensure citizen security, foster institutional collaboration, and allocate resources for public health, utilities, education, climate change mitigation, and food security programs.

## 4. Discussion

Male emigration, inheritance, widowhood, and access to education have contributed to the feminization of livestock farming [21]. Women contribute approximately 43% of all agricultural labor in low- and middle-income countries and play a fundamental role in household food security, dietary diversity, family health, and the conservation and sustainable use of biological diversity [22]. In this study, although women have inherited the farms, accessed higher education, and actively participated in the production system, their recognition is limited by factors described by other authors, such as inequalities based on gender; ethnicity; culture; and social, economic, and political status, which limit their autonomy, decision-making power, and social visibility [23]. Numerous studies confirm the challenges experienced by rural women, regardless of geographic location or ethnic background [12], as described in research conducted in Kenya [24], Indonesia [25], India [26], and the United Kingdom [27]. The most significant barriers have been associated with the following: (a) the lack of financial compensation, the limited recognition of their labor, unequal access to resources, and stereotypical gender roles; (b) limited opportunities for education, mobility, and leisure time; and (c) underrepresentation and limited participation in political and institutional spheres—factors that obscure their contribution to the productivity of agricultural and livestock farms [28]. Additionally, in the present study, women’s opportunities to earn and control financial capital were constrained by the limited recognition of their domestic labor and involvement in secondary activities such as tending to home gardens and caring for backyard animals—tasks that rarely provided economic compensation, in contrast to men’s work.

Recent studies have highlighted the importance of social norms as part of the enabling or inhibiting context for innovative interventions in livestock farming. Gender norms represent the social rules that define what is considered typical and appropriate for women and men to be and do within their societies [29]. These normative frameworks deeply shape how women and men perceive and act upon opportunities in their lives, as well as how institutions design and implement their programs [30]. As a result, women are often perceived as less innovative, and extension services rarely target them [31]. These issues were identified as limiting factors for women in the present study, as they expressed a sense of institutional invisibility and the limited recognition of their role in modern livestock farming. These inequalities have been sustained by various factors, which we grouped into three key sociological perspectives: (a) *The “domination of the masculine”*—This refers to the social construction of meanings and roles that reinforce inequality between men and women. In this dynamic, the male gender holds power and control over the female, a pattern that remains particularly strong in rural areas of Colombia. This is partly due to the influence of tradition and the deeply rooted roles within the structure of rural economies. This social order creates and maintains distinctions between the sexes that are difficult to break and often come to be seen as “natural.” As a result, domination is reinforced, and symbolic violence is expressed through invisible forms of coercion that lead the dominated to accept inequality. This phenomenon is evident in multiple social settings, such as the family, education, and the labor market [32]. (b) *The need for women’s recognition in social contexts*—This has multiple dimensions, reflected in the concept of the “faces of oppression.” In rural areas, these forms of oppression often present more complex and varied expressions: the lack of access to basic resources and education, labor discrimination, exclusion from decision-making, and limited participation in community life, among others. The importance of this perspective lies in how these forms of oppression and the need for recognition are reflected in public policy and social movements. These often assume male dominance in livestock activities and, consequently, promote institutional models and interventions designed primarily for men rather than women. The recognition of women in rural life, therefore, is not just about acknowledging individual women but validating their collective contributions and their efforts to advance together [33]. (c) *Finally*, building on the above ideas, the Economic Commission for Latin America and the Caribbean (ECLAC) has also conducted and promoted similar studies on public policy in the region. These continental-level studies highlight women’s ongoing struggle to serve as heads of households or families while also trying to excel in activities traditionally assigned to men. In this context, women face a double struggle: one in terms of recognition and another in terms of equity [34].

Women livestock farmers are noted for their sensitivity and empathy toward animals, their ethical behavior at work, and their innovative contributions to livestock production [27]. However, persistent gaps in rural environments—such as limited access to information, low literacy rates, and social resistance—remain barriers to the transformation and implementation of more sustainable livestock practices [4,13]. In the natural savanna, women have gradually gained greater involvement in farm management and administrative tasks due to their leadership skills. They are leading productive changes and the implementation of animal welfare practices—especially in the care of cattle, calves, and sick animals—thanks to their caregiving abilities, reflectiveness, and competence, as has also been described in English dairy farms [27]. The patriarchal barriers faced by women in livestock farming can negatively impact the sustainability of production systems and hinder the achievement of long-term Sustainable Development Goals. These effects stem from several key factors: (a) *Underutilization of women’s productive potential*—When women are excluded, skilled labor is lost, along with productivity, household income, and overall system efficiency, particularly due to the lack of access to credit and financial resources. (b) *Dietary diversity and food security*—Women play a key role in food security, as they often prioritize the nutrition of other household members, especially children, and manage household gardens and food supplies. (c) *Environmental impact*—Women tend to be more open to implementing sustainable agricultural practices, such as avoiding agrochemicals, conserving and managing seeds, fostering resilient livelihoods, transforming food systems, and caring for animals in environmentally responsible ways. (d) *Weakened social cohesion and rural abandonment*—When women cannot participate actively and equitably in sustainable policies, it undermines social cohesion and contributes to migration and the depopulation of rural areas. (e) *Loss of local knowledge and cultural diversity*—Women are recognized as proactive catalysts for social change. They are key to passing on traditional knowledge to younger generations and often lead the way in adapting to more demanding markets. (f) *Less resilient production systems*—Excluding women reduces the diversity of ideas, strategies, and management approaches. Their active participation is essential for achieving livestock production systems that are profitable, sustainable, and socially fair [22,33,35]. While new roles for women livestock farmers have emerged, traditional gender norms still limit their practice and contribute to an increased workload—an issue not explored in depth in this study but well documented in other rural research [21].

In Colombia and in many parts of the world, young people involved in livestock farming face multiple challenges and social gaps, which often lead to the abandonment of rural areas and migration to urban centers in search of better living conditions. This process has brought about limitations in generational transfer and the loss of cultural practices in both dairy and beef cattle farming [4]. Negative working conditions for youth can have repercussions on animals or lead to a perception that animal welfare is less important than human well-being [13,36]. In the present study, it was found that both male and female cattle farmers had low incomes, worked under informal labor conditions, and had inequitable access to healthcare services, restricted access to education, and low literacy rates. In addition, they experienced geographic isolation and deficient pre-slaughter infrastructure for cattle. These combined factors discourage young people from pursuing livestock farming as a vocation and reinforce the perception of livestock production as an activity with limited opportunities [4,13].

To recover traditional participatory practices in the livestock sector and promote bidirectional generational transmission—that is, enriching knowledge with new skills without abandoning traditions—several strategies have been proposed to strengthen associative processes and rural policy. These include promoting equal access to education and land ownership, formal employment, and collective work based on the principles of solidarity, discipline, and commitment [4,37]. Likewise, fostering a sense of belonging to the territory through the social appropriation of local culture, the development of organizational skills, and the political inclusion of rural youth is encouraged, along with the exchange of knowledge and experiences [37]. In Colombia, there are participatory education and co-creation programs promoted by the private sector that have had transformative effects on rural youth, such as the “Lab Campesino” [37] and “Heirs of the Tradition” programs [4]. These initiatives aim to promote technical literacy in livestock production, strengthen cultural heritage, and empower new generations by stimulating creativity, improving access to technology, and fostering the transmission, co-creation, and sharing of knowledge. These efforts aim to enhance the competitiveness of livestock enterprises and improve interpersonal and social skills [4,37]. However, a major limitation of these programs was the underdevelopment of digital capacities due to limited internet access. In addition, learning continuity was hindered by armed conflict and widespread violence. Gender inequalities also restricted women’s participation in educational programs, livestock practices, and political engagement—as observed in our study and in other geographical and cultural contexts [38].

Society is increasingly demanding more sustainable production systems that protect ecosystem health and resilience, animal welfare, and the quality of human life [22,39], along with the concept of *social license to operate* [40]. A social license to operate (SLO) is an intangible, unwritten, and non-legally binding social contract through which a community grants or denies permission for an industry to operate [6,40]. Animal welfare serves as a guarantor for society, granting livestock systems a “social license to farm,” since scientific evidence has shown that its implementation has positive effects on animals, enhances the human–animal relationship, improves productivity, and increases meat quality [9,39]. In this context, the pre-slaughter stage represents a critical point in the welfare of animals and has been subject to ethical scrutiny by consumers. In Colombia, several studies have documented that livestock transportation is not specialized; slaughterhouses are located far from production centers; and geographic conditions, road infrastructure, and cold chains are deficient, among other issues [41,42]. To mitigate these challenges, the establishment of modern regional slaughterhouses has been proposed as a central strategy to promote both domestic consumption and international markets by fostering potential links between producers and specialized markets [41].

An agricultural modeling study conducted by researchers in the department of Vichada found that transitioning from traditional systems to systems with improved pastures would require the exclusive allocation of 2000 hectares of land for livestock farming and the hiring of approximately 19 permanent workers and an additional group of temporary workers during the periods of April–July and November–December [14]. However, the producers involved in this study faced several challenges and limitations, such as lack of professional technical support, low borrowing capacity, limited access to financial capital, poor business management, a shortage of labor, inadequate infrastructure, a lack of confidence in investments due to the presence of illegal armed groups, and weak producer associations. These factors significantly limit the feasibility of implementing such changes, especially for small-scale farmers. On the other hand, in other departments of the Orinoquia region, production systems are being transformed through the introduction of improved pastures, perennial crops (such as oil palm, cacao, and cashew), and mechanized crops like rice, corn, and soybeans; the development of fattening pig production systems; and oil extraction activities [14]. While these developments have stimulated the region’s economy, they have also had negative impacts on the environment and regional security.

Today, the most demanding markets for fresh beef are looking for more sustainable farming systems—those that protect the environment, promote animal welfare, and improve people’s quality of life. In these markets, animal welfare is not just an ethical issue but also an essential part of sustainability. Consumers and society expect better practices, and many countries have made this a key part of their food production system, following the “farm to fork” approach. This has been especially true in major beef-producing countries like Uruguay, Brazil, and Argentina and even in emerging markets like Chile, where 70–80% of farms are family-run operations [39]. For this reason, it is worth exploring the potential of the Vichada savannas in Colombia, which could be ideal for sustainable agriculture that supports both people and the environment—following the “One Health, One Welfare” approach. The region’s ecosystem services are seen as a big advantage. Pasture-based cattle farming is especially valued for producing “natural” beef—free from additives, routine antibiotics, growth hormones, and chemical residues. This gives the meat extra value in the eyes of careful consumers [43]. Many consumers also see pasture-raised cattle as more ethical and natural than animals raised in confinement. Animal welfare is now seen as a sign of good meat quality [43]. But to move toward this model, Colombia needs good public policies, support from local and national governments, training in sustainable farming, land security, better slaughter infrastructure, and access to premium markets with special labels and certifications [39,44]. Colombia already has some programs and policies to support more sustainable livestock farming. These include voluntary standards that certify environmentally friendly beef and national strategies to protect forests, restore ecosystems, and make cattle farming more sustainable—using systems like silvopasture and rotational grazing [45]. However, more research is still needed to find the best approaches for the Colombian savanna regions.

Colombia has responded to these challenges by updating its legal framework regarding animal welfare. This includes the implementation of welfare practices on farms [46] and their certification [47], regulations on animal transport [48], and guidelines for the slaughter of cattle, buffalo, and pigs [49], as well as the training and certification of livestock transporters and handlers [48]. However, as highlighted in this study, further improvements are needed in road infrastructure, the development of specialized transport systems, incentives for the livestock sector, and enhancements to the quality of life of producers.

Despite advances in legislative frameworks promoting the implementation of primary production practices, animal welfare, and the control of high-impact animal diseases relevant to international trade, farmers in this study—as well as those in other parts of the world—perceive these requirements as, at times, unfair. They often represent an administrative, economic, and social burden that is insufficiently acknowledged by legislators and lack incentives for those who comply [50]. Furthermore, these regulations are typically imposed in a top-down manner and overlook the limitations small-scale producers face in meeting such standards. Similarly, resistance to change and the low adoption of innovations by older producers may be linked to a strong attachment to traditional practices rooted in collective heritage, experiential knowledge, and the fear of losing culturally embedded wisdom [51]. These two challenges could be addressed through the development of policies that promote the active participation of all stakeholders and responsible governance. Such governance should be based on empowering producers, strengthening farmer associations, and encouraging the active involvement of producers in policymaking processes. This approach would enhance understanding and improve both animal health and welfare—elements previously discussed by the authors in the first part of this study [7].

### Limitations

The mixed-methods approach (qualitative and quantitative) used in this study aimed at identifying the maximum variation; therefore, the results do not reflect how widespread the issues described are among cattle farms in part of the Colombian natural savanna (Vichada department).

## 5. Conclusions

Cattle farmers have a direct impact on both animal welfare and productivity. The relationships between farmers and animals are multifactorial, highlighting the need for studies that approach this topic comprehensively. The continuity of livestock culture and the improvement of human–animal relationships can be achieved through the development of a culture of continuous learning, tailored to the social and cultural conditions of the producers.

It is essential to promote bidirectional knowledge transfer, in which family structures and innovation adoption are fundamental. Equally important is the improvement of socioeconomic conditions, to ensure that new generations of farmers remain in the region under more equitable social and labor relations—relations that are sensitive to gender-based cultural transitions and open to the adoption of new technologies while still preserving their cultural and social values.

The empowerment and autonomy of women must be strengthened through actions aimed at fostering self-esteem, self-respect, self-confidence, and self-sufficiency. Additionally, innovative approaches are needed to create income-generating opportunities and to develop political interventions that encourage women’s effective participation.

Although our study did not evaluate the socioeconomic, political, and environmental differences among cattle farmers based on their geographic location in the flat and irregular high plains (*flat and dissected Altillanuras*) of the Orinoquia region in the department of Vichada, future research should explore these factors. They may influence farmers’ perceptions of human, animal, and environmental welfare, as well as the sustainability of their production systems. This is important considering that their economic activities and settlement patterns are shaped by geographic characteristics. In the flat high plains, extensive cattle ranching under native pasture conditions predominates, with more dispersed settlement patterns. In contrast, the irregular high plains allow for greater economic diversity, including agriculture, fishing, and commercial activities in more densely populated urban centers.

Finally, it is necessary to integrate social sciences and the humanities in order to better understand human–animal relationships and to promote more inclusive and sustainable agricultural policies.

## Figures and Tables

**Table 1:** Demographics of participants in quantitative (survey = 65) and qualitative studies (focus group participants = 24; interview participants = 11) on perceptions related to One Welfare approach, La Primavera, Vichada.

Characteristics		Survey n (%)	Interviews n (%)	Focal Groups n (%)
Gender	Male	59(90.8)	10(90.9)	14(58)
	Female	6(9.2)	1(9.1)	10(42)
Age	22–32	2(3.1)	1(9.1)	1(4.2)
	33–43	12(18.6)	3(27.3)	9(37)
	44–54	24(36.8)	4(36.3)	6(25.2)
	55–65	21(32.2)	3(27.3)	5(21)
	66–76	6(9.3)	“_”	3(12.6)
Level of education	No education	10(15.5)	“_”	4(16.8)
	Primary	45(68.9)	2(18.2)	10(42)
	Secondary	9(13.9)	1(9.1)	4(16)
	Post-secondary	1(1.7)	8(72,1)	6(25.2)
Years of experience	8–18	9(13.9)	1(9,1)	“_”
	19–29	10(15.3)	5(45.4)	1(4)
	30–40	30(45,2)	4(36.4)	13(54)
	41–51	14(22.4)	“_”	5(21)
	52–62	2(3.2)	1(9.1)	5(21)

**Table 2:** Description of qualitative and quantitative results related to Theme 1: transformation factors in livestock activities.

Topics and Subtopics	Quotes That Reflect the Subtopic	Quantitative Results That Support the Subtopic
Subtopic 1: Gender Cultural Transitions		
a. Change in the role of women in livestock farming due to inheritance and widowhood	[P5_GR2M]: “... My link to livestock also comes from family, from inheritance... over time, you see how your father manages the cattle... or you go to work in the plains, and you grow fond of livestock.”	Quantitative: 90.8% of farms are managed by men (*n* = 59), 9.2% by women (*n* = 6)
b. Widowhood	[P11_GR2M]: “When my husband died and I was left with the farm, everyone told me: sell it! Because you’re a woman... but I said to myself: how can I not be capable... and I took over the farm.”	Quantitative: 95% married producers (*n* = 62), 5% single or widowed (*n* = 3)
c. Access to education	[P7_GR2M]: “They taught me from a young age to be passionate about livestock. I studied at university... I’m an agribusiness and livestock manager.”	Livestock producers have begun pursuing post-secondary education (Table 1)
Subtopic 2: Lack of Generational Replacement		
a. Sociodemographic changes	[P30_GR1H]: “The older people are dying out, and even if they say my father and grandfather were farmers, the reality is the activity is being abandoned...”.	Age range of farmers: 22–43 (18.5%), 44–54 (36.9%), 55–65 (32.3%), 66–76 (9.2%)
b. Lack of opportunities for young people in rural areas	[P28_GR3]: “This is another problem. Young people study but don’t want to return to the plains because there are no job opportunities.”	Only 3.1% (*n* = 2) of farmers are between 22 and 32 years old
c. Access to education	[P122_INTERH] “…Schools are a long way from farms. Parents send their children to schools for a week or two so they can study.”	Low education level (Table 1)
d. Livestock	[P119_INTERH] “Traditional farmers love this lifestyle but would live better with more government support—and that’s not happening.” [P120_INTERH] “… Ranching is a job that requires a lot of sacrifice, specially working in in the fields, because you get up very early, and have to do a lot, and return exhausted and have a little time to be with the family…”	93.8% (*n* = 61) consider livestock farming important for society
The song of the plains	[P17_GR1H]: “The cattle driving song is a part of culture and it must be protected. One thing is the song during milking, and another is the song so that the cattle calms down.”	93.8% (*n* = 61) consider livestock farming important for society

**Table 3:** Description of qualitative and quantitative results related to topic 2: changes in relationship between farmers and animals.

Subtopics	Quotes That Reflect the Subtopic	Quantitative Results That Support the Subtopic
a. Implementation of new production practices and innovations	[P28_INTERH] “There have been changes. Due to this generational shift, my father managed everything extensively, and now that I’m a veterinarian, I bring a different perspective—not only focused on the economy but also considering social, environmental, cultural, and political aspects.” [P36_GR2M]: “Today, women are taken into account more, because before we weren’t seen at all—only men [...] now I manage the farm, and I haven’t let it decline.”	
b. Adoption of innovations	[P6_GR3I]: “Those who propose big changes are not locals, they are outsiders who motivate producers to improve and adopt new practices and technologies like artificial insemination, pasture management, and more.”	A total of 18.5% (*n* = 12) of farmers associate changes with adopting new technologies for animal management and 32.3% (*n* = 21) with changes in production culture.
c. Improvement in animal welfare practices	[P65_INTERH]: “My mother handled milking and taming the cows; my father only helped when the cow was aggressive or hard to milk. Generally, taming is done by women—they’re gentle, they care…” [P108_INTERH]: “When you herd cattle, the way you whistle or shout at them affects their behavior. It’s easier to handle cattle used to humans, especially if treated well—it creates a special connection. Poor handling leads to aggressive animals, because poor handling has consequences.”	Among female farm administrators (*n* = 17), 72.3% carry out cattle herding and household management activities.

**Table 4:** Description of qualitative and quantitative results related to topic 3: conditions for change.

Subtopics	Quotes That Reflect the Subtopic	Quantitative Results That Support the Subtopic
a. Invisible and undervalued contribution of women	[P71_INTERH]: “After milking, women do household chores, cook for the family and workers, clean the house, take care of the children, handle backyard animals (chickens, pigs, sheep), and care for the garden.”	A total of 67.7% (*n* = 44) of women receive no income, 23.1% (*n* = 15) earn between 0.5 and 1 minimum wage, 6.2% (*n* = 4) earn 1–2 minimum wages, and 3% (*n* = 1) earn over 2 minimum wages.
b. Discriminatory gender norms	[P8_GR2M]: “One day my dad mistreated my mom saying: ‘You’re a woman, and women belong in the kitchen.’ I told him: ‘No, dad, women are just as important—ranching is not a gender issue, it’s about love and administrative skill, which we women have!’” [P30_GR3]: “Gender issues evolve culturally. Llaneros think women belong in the kitchen and home. But this has been changing somewhat with more education and training opportunities for women.”	A total of 90.8% (*n* = 59) of farms are managed by men and 9.2% (*n* = 6) by women.
c. Lack of institutional recognition	[P40_GR2W]: “I would like to see institutions offering training and guidance for women and they should call us together […], women are very conscientious, hard-working, and we like to learn.”	
d. Low income and poor access to financing	[P19_GR3]: “Another problem is financial—adopting changes and technology is expensive. A proper corral with chute costs a lot. I told my brother to build one, and he said he’d have to sell a cow, which would leave him with nothing.”	A total of 52.3% (*n* = 34) have no formal employment link, 12.3% (*n* = 8) have formal contracts (written), and 35.4% (*n* = 23) have verbal contracts. A total of 46.1% (*n* = 30) have no income other than ranching, 47.7% (*n* = 31) have income from agriculture or commissions, and 6.2% (*n* = 4) have income from general services. Reasons for insufficient income: 52.3% (*n* = 34) high cost of living, 15.4% (*n* = 10) low salary, 32.3% (*n* = 21) other cost-of-living issues.
	[P112_INTERH] “Credit for farmerss is limited, aimed only at those who meet bank requirements. There’s been no real government support for projects, infrastructure, or machinery, so everyone works individually—no incentives.”	A total of 66.2% (*n* = 43) have never accessed bank credit, while 33.8% (*n* = 22) have accessed it at least once.
e. Institutions’ basic sanitation	[P110_Int] “…Ranching is seen as a second or third-class activity. Being an ‘agro worker’ still seems very rustic and primitive.” [P123_INTERH] “Without electricity, we’re nothing. If the state really invested in this, the region would thrive. Go to Puerto Carreño—people don’t even have electricity.”	A total of 6.9% (*n* = 11) have no electricity, 81.5% (*n* = 53) use solar energy, and 1.5% (*n* = 1) use a solar + gasoline generator. Potable water source: 64.6% (*n* = 42) from deep wells, 13% (*n* = 20) from natural springs, 15.4% (*n* = 10) from streams. Cooking energy source: 60% (*n* = 39) firewood, 30.8% (*n* = 20) gas + firewood, 9.2% (*n* = 6) only natural gas. Waste disposal: 56.9% (*n* = 37) use toilets with septic tanks, 41.6% (*n* = 27) have more than one toilet, 1.5% (*n* = 1) defecate in the open.
f. Information and communication technology ICT	[P118_INTERH] “Internet signal is very poor in some regions.”	A total of 78.5% (*n* = 51) have mobile phones, 12.3% (*n* = 8) have access to radio/TV/internet, and 9.2% (*n* = 6) have no access.
g. Infrastructure—lack of roads	[P33_GR1H]: “…What’s the point of raising well-kept cattle with shade and water if, when transporting them inland, we lose everything due to bad roads. Without proper access, all the effort is lost.”	No local slaughterhouses in the region, 100% (*n* = 65) do not sell cattle by scale, 86.2% (*n* = 56) have wooden corrals, 9.2% (*n* = 6) have cement corrals, and 4.6% (*n* = 3) have none.
	[P5_GR1M]: “It’s a struggle to move animals—even loading them is hard without proper docks—we feel bad and so do the animals. We don’t get state licenses to unload them. It’s a horrible—for us and for the animals, and it generates a lot of cost”	A total of 92.3% (*n* = 60) of farms do not have a cattle handling pen.
h. Security	[P117_INTERH] “We’re at the mercy of cattle theft and no one helps. The state doesn’t intervene. Even when thieves are caught, they’re not prosecuted. There’s no peace or security anymore, everything is affected by insecurity...”	Security perception: 15.4% (*n* = 10) good, 49.2% (*n* = 32) regular, 35.4% (*n* = 23) bad. A total of 100% (*n* = 65) report the main security issues as cattle theft and extortion.
i. Resistance to change	[P4_GR3I]: “I’m a Llanero (Farmer) and I know change is needed, but to what extent does new technology strip us of our essence? Institutions should respect and consider our identity...”	The importance of livestock activity for farmers is based on 32.3% (*n* = 21) keeping the plains tradition alive, 58.5% (*n* = 38) on local economic development, 3.1% (*n* = 2) on food production, and 6.1% (*n* = 4) on the generation of new knowledge.
	[P3_GR1H] “For me, it’s a complex issue because right now we have to apply many vaccines each year, and the government requires them—three a year—but we have to apply two more. [...] We know that we have to take care of livestock; so I don’t know how to interpret animal welfare. That’s like a contradiction.” [P124_INTERH] “...Traditional livestock farmers vaccinate because they have the authority pressuring them, or else they wouldn’t vaccinate [...] Institutions have not been able to get livestock farmers to integrate into their knowledge the usefulness of vaccination for controlling officially controlled diseases.”	

**Table 5:** Description of qualitative and quantitative results related to Theme 4: challenges and opportunities for improvement.

Subtopics	Quotes That Reflect the Subtopic	Quantitative Results That Support the Subtopic
Empowerment of women and rural youths	[P27_GR3] “As for gender policies, these require public institutions to hire women, but on the other hand, today there are women in the region who are highly qualified academically and have experience, so it’s no longer necessary to hire them from other departments...”	A total of 100% (*n* = 65) of the livestock farmers report that the main credit institution is the Agricultural Bank.
	[P37_GR2M]: “I think it would be important to include young people in the new changes, because we need to integrate them more so they fall in love with the livestock culture. We should invite our children to participate.”	
	[P115_INTERH] “... Credit, which is the only option a farmer has, apart from his capital, selling cows to acquire technology, is not possible because it would diminish his working capital...”	
Integrative pedagogical methodologies	[P30_GR3]: “… I think life in the plains has changed somewhat as there are more opportunities for education and training for women and young people. So I think what we need to achieve are more mechanisms so that people can get an education, so that they can get training, and develop.”	A total of 1.5% (*n* = 1) of the farmers acquire their knowledge through formal education, 98.5% (*n* = 64) through tradition and the exchange of knowledge.
	[P1_INTERM] “As I’m a veterinarian, if there’s any problem with the animals, I go and examine them; if any procedure needs to be performed, it’s done. With vaccinations, we also have to make sure both cycles are completed.”	
Access routes and commercialization	[P116_INTERH] “… the conditions of the roads and pathways are very poor. If you produce very good livestock, you don’t have the conditions to market them, because unfortunately, there’s no support from the national government for the countryside, in terms of roads and communications…” [P14_GR3I]: “I believe that the change in livestock transportation has been improving; we’ve gone from transporting them by river to transporting them by land [...]. Previously, there were no bridges, and livestock had to be swum across the river, and that was a risk for the animals, for the plainsmen, and the horses. Now we have some bridges, but many roads are still missing, and transport times are very long.”	There are two slaughterhouses for domestic consumption in the department. During the summer, cattle are transported on foot and by road by truck and during the winter, on foot or by boat on the river. Fifty percent (*n* = 2) of the municipalities in the department of Vichada have a slaughterhouse for domestic consumption. One hundred percent (*n* = 65) of the access roads to the farms are unpaved.
Governance	[P34_GR3]: “…When social security breaks down, it affects investment, and if there’s no investment, there’s no employment, no doctor, and then you have to go out to the city for just about anything, and that complicates your life. So the lack of security does us a lot of harm [...] and thus, backwardness develops in the region and the quality of life of the residents weakens.” [P29_GR3]: “For me, there’s no institutional coordination, because each one has its own guidelines and they don’t work in an integrated manner. Coordination between the public and private sectors would be very important to provide better services, foster partnerships, and have the support of veterinarians.”	

## Data Availability

The data that support the findings of this study are available on request from the corresponding author. The data are not publicly available due to privacy or ethical restrictions.

## Supplementary Materials

The following supporting information can be downloaded at https://www.mdpi.com/article/10.3390/ani15142139/s1, Guide S1: Interview guide.
