# Supplementary material for: Natural Savanna Systems Within the “One Health and One Welfare” Approach: Part 2—Sociodemographic and Institution Factors Impacting Relationships Between Farmers and Livestock
Source: Animals (Basel). 2025 Jul 19;15(14):2139. doi: 10.3390/ani15142139 (PMC12291747; doi:10.3390/ani15142139)
Supplement: Supplementary file 1 [file animals-15-02139-s001.zip › animals-3725300-supplementary.pdf]

**Natural Savannah Systems Within the "One Health and One Welfare" Approach: Part 2-Sociodemographic, biogeographical and institution factors impacting relationships between farmers and livestock**

**Semi-structured interview guide**

| Base Question | Topics Related to the Question |
|---|---|
| **1. What is the role of women in livestock farming? Are women considered key players in herd productivity and development?** | Women in livestock; gender relations in productive development; stereotypes (e.g., "women are not capable," "it's hard work") |
| **2. What is the predominant educational level of producers? Could we analyse this issue?** | Education in the agricultural sector; educational opportunities; interests; educational offerings; territorial educational conditions |
| **3. What is the role of the family (mother, father, and children) in livestock production activities?** | Role of the family: nuclear and extended family; collaborative work; livestock farming and child-rearing |
| **4. From your point of view, how would you assess the food sovereignty of livestock-raising families in the region?** | Home gardens, interest in growing food, seed availability, traditional practices, methods, species variety |
| **5. What factors are associated with the quality of life of livestock farmers?** | Clothing, housing, public services, internet, phone service, education, healthcare — conditions influencing well-being |
| **6. What is the predominant production model in livestock farming?** | Social relations of production: means of production, land ownership, who works the land, who owns it — predominant model in the region |
| **7. How are daily activities carried out on a livestock farm? Has it always been this way?** | Start time, working hours, free time/leisure, participants by time, spaces and their meaning; plains songs; tool-making; use of technology; |
| 8. **Do you think the producers find happiness in their activities?** | Emotional states linked to livestock farming — joy, anger, love for the work |
| **9. Do you believe livestock farming has specific recognition in various areas of social life?** | Environmental, economic, productive, political, cultural, and social recognition |
| **10. How do producers acquire new knowledge and technical skills?** | Technologies, production methods, commercial opportunities, approaches to shared challenges |

| | |
|---|---|
| **11. How is the community and social organization of livestock farmers structured?** | Como es ese tejido, como se organizan los liderazgos, la federación tiene a todos los ganaderos, como se apropian de estas organizaciones, como es su liderazgo, se organizan todos, como es esa estructura |
| **12. Do you believe livestock farming in the region is a safe activity? What are the safety factors?** | Physical and territorial security, public order, violence, land invasions. |
| **13. Are you knowledgeable about training in animal welfare?**<br>**14. Do you believe animal welfare freedoms affect livestock farming practices?** | **Freedoms:** Good nutrition, good health, adequate housing, natural behaviour;<br>**Nutrition:** Natural savannah;<br>**Health:** Treatment and disease management;<br>**Housing:** Savannahs as shelter, heat stress, infrastructure;<br>**Behaviour:** Human-animal interactions |
| **15. What is your opinion on environmental education aimed at livestock farmers?** | Formal or informal education; role of institutions and private sectors |
| 16. **What is your opinion on the relationship between livestock farming and the territory's flora and fauna?** | Farm environments, native forests, rivers, streams, conservation areas, waste, species, |
